# Whole genome sequencing for the molecular characterization of carbapenem-resistant *Klebsiella pneumoniae* strains isolated at the Italian ASST Fatebenefratelli Sacco Hospital, 2012–2014

**DOI:** 10.1186/s12879-017-2760-7

**Published:** 2017-10-10

**Authors:** Sara Giordana Rimoldi, Bernardina Gentile, Cristina Pagani, Annamaria Di Gregorio, Anna Anselmo, Anna Maria Palozzi, Antonella Fortunato, Valentina Pittiglio, Anna Lisa Ridolfo, Maria Rita Gismondo, Giuliano Rizzardini, Florigio Lista

**Affiliations:** 1 Laboratory of Clinical Microbiology, Virology and Bioemergencies, ASST Fatebenefratelli Sacco, University Hospital “Luigi Sacco”, Via G.B. Grassi 74, 20157 Milan, Italy; 2 Scientific Department Army Medical Center, Via Santo Stefano Rotondo 4, 00184 Rome, Italy; 3 Infectious Diseases Department, ASST Fatebenefratelli Sacco, University Hospital “Luigi Sacco”, Via G.B. Grassi 74, 20157 Milan, Italy; 4 Infectious Diseases Department, I Division, ASST Fatebenefratelli Sacco, University Hospital “Luigi Sacco”, Via G.B. Grassi 74, 20157 Milan, Italy

**Keywords:** Carbapenem-resistant *Klebsiella pneumoniae*, Whole-genome sequencing, Bacteria epidemiology

## Abstract

**Background:**

The emergence of carbapenem-resistant *Klebsiella pneumoniae* strains is threatening antimicrobial treatment.

**Methods:**

Sixty-eight carbapenemase-producing *K. pneumoniae* strains isolated at Luigi Sacco University Hospital-ASST Fatebenefratelli Sacco (Milan, Italy) between 2012 and 2014 were characterised microbiologically and molecularly. They were tested for drug susceptibility and carbapenemase phenotypes, investigated by means of repetitive extra-genic palindromic polymerase chain reaction (REP-PCR), and fully sequenced by means of next-generation sequencing for the in silico analysis of multi-locus sequence typing (MLST), their resistome, virulome and plasmid content, and their core single nucleotide polymorphism (SNP) genotypes.

**Results:**

All of the samples were resistant to carbapenems, other β-lactams and ciprofloxacin; many were resistant to aminoglycosides and tigecycline; and seven were resistant to colistin. Resistome analysis revealed the presence of *blaKPC* genes and, less frequently *blaSHV*, *blaTEM*, *blaCTX-M* and *blaOXA*, which are related to resistance to carbapenem and other β-lactams. Other genes conferring resistance to aminoglycoside, fluoroquinolone, phenicol, sulphonamide, tetracycline, trimethoprim and macrolide-lincosamide-streptogramin were also detected. Genes related to AcrAB-TolC efflux pump-dependent and pump-independent tigecycline resistance mechanisms were investigated, but it was not possible to clearly correlate the genomic features with tigecycline resistance because of the presence of a common mutation in susceptible, intermediate and resistant strains. Concerning colistin resistance, the *mgrB* gene was disrupted by an IS5-like element, and the mobile *mcr-1* and *mcr-2* genes were not detected in two cases. The virulome profile revealed type-3 fimbriae and iron uptake system genes, which are important during the colonisation stage in the mammalian host environment. The in silico detected plasmid replicons were classified as IncFIB(pQil), IncFIB(K), ColRNAI, IncX1, IncX3, IncFII(K), IncN, IncL/M(pMU407) and IncFIA(HI1). REP-PCR showed five major clusters, and MLST revealed six different sequence types: 512, 258, 307, 1519, 745 and 101. Core SNP genotyping, which led to four clusters, correlated with the MLST data. Isolates of the same sequencing type often had common genetic traits, but the SNP analysis allowed greater strain tracking and discrimination than either the REP-PCR or MLST analysis.

**Conclusion:**

Our findings support the importance of implementing bacterial genomics in clinical medicine in order to complement traditional methods and overcome their limited resolution.

**Electronic supplementary material:**

The online version of this article (10.1186/s12879-017-2760-7) contains supplementary material, which is available to authorized users.

## Background

*Klebsiella pneumoniae* bacteria are normal gastrointestinal flora but, particularly in people with weakened immune systems and/or debilitating diseases, can also cause severe infections whose management has been complicated by increasing antimicrobial resistance over the last 20 years. Global surveillance studies carried out during the 2000s showed that 20–80% of *K. pneumoniae* isolates were resistant to first-line antibiotics, including the cephalosporins, fluoroquinolones and aminoglycosides [1–3]. However, the recent worldwide spread of strains that are resistant to carbapenems is even more worrying because these antibiotics are often the last line of effective treatment of the infections caused by multidrug-resistant *K. pneumoniae* [4]. Various carbapenem-resistance mechanisms have so far been identified, but the most frequent is related to the production of *K. pneumoniae* carbapenemase (KPC), the expression of which is largely due to the plasmid-derived *blaKPC* gene, although some cases of chromosomally integrated *blaKPC* genes have been described in the literature [5–7].

The currently few treatment options for carbapenem-resistant *K. pneumoniae* (CRKP) infections include polymyxins, tigecycline, aminoglycosides and fosfomycin, but their use is limited by concerns about their efficacy and safety [8], and there are increasing reports of resistance to colistin and tigecycline [9, 10]. In the absence of aggressive infection control, CRKP can easily and rapidly spread, which explains why they have been responsible for outbreaks of infection within and between healthcare facilities [11]. Since 2010, Italy has seen a remarkable increase in the occurrence of CRKP, which greatly contributed to the epidemic spread of carbapenem-resistant Enterobacteriaceae (CRE) recorded by a countrywide cross-sectional survey, the results of which were published in 2013 [12].

Methods of discriminating and characterising different *K. pneumoniae* isolates are essential in order to be able to direct the targeting of infection control resources. Traditional systems based on phenotypes (serotypes, biotypes or antibiograms), and molecular methods such as multi-locus sequence typing (MLST) and repetitive extra-genic palindromic polymerase chain reaction (REP-PCR) have long been used, but may not provide sufficient resolution because of the high degree of clonality of *K. pneumoniae* [13]. Following improvements in sequencing technologies, whole-genome sequencing (WGS) is now well placed to become the gold standard for bacterial typing as it can define the complete genomic structure of a pathogen [14, 15] which, in the case of *K. pneumoniae*, is one circular chromosome and a variable number of small and large plasmids, the primary sources of virulence and resistance [16–18].

We fully sequenced 68 *K. pneumoniae* strains isolated at Luigi Sacco University Hospital-ASST Fatebenefratelli Sacco (Milan, Italy) that had previously been tested for drug susceptibility and carbapenemase phenotyping, and investigated by means of REP-PCR. In order to compare and integrate the microbiological, phenotypic and genotypic data with the in silico and whole-genome analyses, we used web tools and bioinformatics software to characterise the genotypic, resistance and virulence traits of *K. pneumoniae* molecularly. Starting from the WGS data, we defined the sequence type (ST) strains using in silico MLST (the gold standard for *Klebsiella* genotyping), determined correlations among the samples by analysing the core single nucleotide polymorphisms (SNPs), and extracted molecular data that are important for clinical and epidemiological purposes.

## Methods

### Testing susceptibility and detecting resistance phenotypes

Sixty-eight *CRKP* strains were collected from sixty-eight patients admitted to various wards of Luigi Sacco University Hospital-ASST Fatebenefratelli Sacco (Milan, Italy) between 2012 and 2014. The bacterial strains were obtained from routine microbiological cultures of clinical samples (e.g. urine, wound exudates, bronchial secretions, blood or peritoneal fluid) collected from the medicine (32.4%), infectious diseases (29.4%), surgery (21%), and other wards (8.8%), and the intensive care unit (5.9%),.

The species and their antimicrobial susceptibility were determined using the Vitek 2 automated system (BioMérieux, Marcy l’Etoile, France): resistance to carbapenems was established by interpreting the results of the antimicrobial susceptibility test on the basis of the breakpoint criteria of the European Committee on Antimicrobial Susceptibility Testing [19], and susceptibility to colistin and tigecycline was verified using the E-test (BioMérieux). Carbapenemase phenotyping was carried out using the KPC + MBL Corfirm ID kit (Rosco Diagnostica A/S, Taastrup, Denmark) as recommended by the manufacturer.

### DNA extraction and quantification

Genomic DNA was extracted using an automated DNA/RNA extraction system (Maxwell16, Promega, Madison, WI, USA). The quality, quantity and purity of the DNA were determined using an agarose gel NanoDrop 8000 spectrophotometer (Thermo Fisher Scientific, Waltham, MA, USA) and TBS-380 Mini-Fluorometer (Topac Inc., Cohasset, MA, USA).

### Rep-PCR

REP-PCR, which studies intergenic repetitive coding sequences scattered across the genome, was carried out using a DiversiLab *K. pneumoniae* fingerprinting kit (BioMérieux). The amplification products were detected and sized using microfluidic Lab-Chips and the Agilent 2100 Bioanalyser (Agilent Technologies, Diegem, Belgium). The DNA band patterns were analysed by means of DiversiLab web-based software using Pearson correlation coefficient pairwise pattern matching and the unweighted pair group method with arithmetic mean (UPGMA) clustering algorithm to create dendrograms.

### WGS and in silico data analysis

The samples were fully sequenced using next-generation sequencing on the Illumina MiSeq platform (San Diego, CA, USA). The Nextera XT DNA protocol was followed using 1–1.5 ng of starting DNA and a v3 kit (600 cycles). The paired-end reads were barcode de-multiplexed into separate samples, and quality checked in order to remove adapter sequences and bases with a quality score of <25 using FastQC and Sickle software [20, 21]. The reads were de novo assembled into contigs using Abyss-pe, version 1.5.2 (k parameter = 63) [22], with contigs of >500 bp being selected by means of an ad hoc script, and kept for further analysis.

The in silico MLST analysis was made by comparing the whole-genome sequences against the *Klebsiella pneumoniae* Pub MLST database (http://bigsdb.pasteur.fr/klebsiella/klebsiella.html) in order to assign allelic numbers to all ST loci. Phyloviz software based on the goeBURST algorithm was used to visualise the evolutionary relationships among the isolates [23, 24].

The resistome of antimicrobial resistance genes was analysed using ResFinder-2.1 software (default identity thresholds [ID] 98%) provided by the Center for Genomic Epidemiology (http://www.genomicepidemiology.org) and the resources of the Pasteur MLST *K. pneumoniae* database, and by locally running the BLAST program. Previously described genes relating to AcrAB-TolC efflux pump-dependent and pump-independent tigecycline resistance mechanisms (*acrR*, *ramR*, *marR*, *soxR*, *lon*, *rpsJ* and *rpoC)* [25, 26], and chromosomal (*mgrB, pmrA/pmrB, phoP/phoQ*) and plasmidic (*mcr-1* and *mcr-2*) colistin resistance-related genes [27, 28] were analysed in order to elucidate the possible determinants of tigecycline and colistin resistance. In the case of *blaKPC*-negative strains, the possible association between carbapenem resistance and the loss or alteration of outer membrane porins was investigated by studying the *ompK35* and *ompK36* genes.

The virulome of virulence factors was analysed using the Pasteur MLST *K. pneumoniae* database. The investigated genes were those encoding for 3 fimbrial adhesins (mrk operon), those involved in iron acquisition systems (irp2, fya, kfu, ybt), and the *wzi* gene allele that allows the determination of the Klebsiella capsular type.

PlasmidFinder-1.3 (ID 98%; http://www.genomicepidemiology.org) was used to define the content of the plasmid replicon types, and PROVEAN was used to predict the biological impact of an amino acid substitution or indel on protein function [29]. The Mauve program was used to obtain multiple DNA or protein sequence alignments [30]. SNPs were detected using the kSNP v2.1.2 program (*k*-mer = 21), which defines an SNP locus as an oligo of length *k* surrounding a central SNP allele [31]. The maximum likelihood tree based on the common SNPs detected in all of the genomes (core SNPs) was visualised using Dendroscope v3.2.10 software [32].

## Results

### Traditional microbiological and molecular analyses

**Fig. 1:**
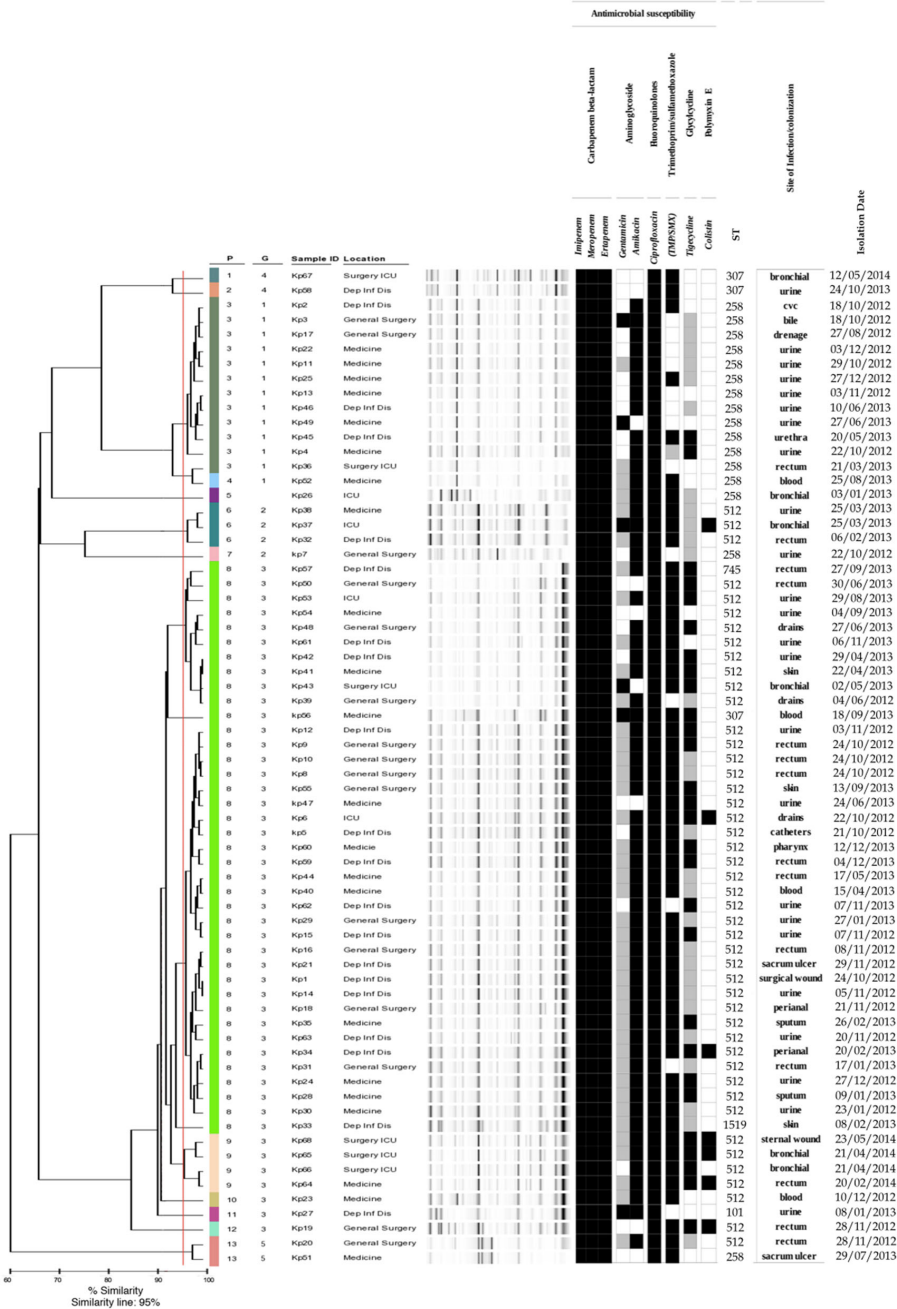
Dendrogram analysis, virtual gel image of REP-PCR fingerprint patterns and antibiotic resistance profiles of 68 carbapenem-resistant *K. pneumoniae* strains. Strains were considered to be genetically related if their fingerprint band patterns were ≥95% similar. Strains resistant, intermediate and sensitive to antibiotics are shown in black, gray and white, respectively. P: Pattern, indicates samples with indistinguishable electrophoretic profile. G: Group, indicates isolates differing for less than three electrophoretic bands

Figure 1 shows the antimicrobial susceptibility findings and clonal relationships between the isolates obtained by means of traditional methods. The 68 isolates showed resistance to all β-lactams and ciprofloxacin, and all but one were carbapenemase producers. Almost 90% were resistant to at least one of the two aminoglycosides tested; 47% were resistant to tigecycline; seven samples (10.30%) were resistant to colistin; and two (3.1%) were intermediate/resistant to all of the tested antibiotics (Additional file 1: Table S1).

The use of REP-PCR revealed 13 DiversiLab patterns that clustered into five groups. The most prevalent was cluster 3 (*n* = 46 samples) followed by cluster 1 (*n* = 13), 2 (*n* = 4), 4 (*n* = 2) and 5 (*n* = 2). One isolate was a singleton (Fig. 1).

### Whole-genome sequencing and in silico data analysis

**Fig. 2:**
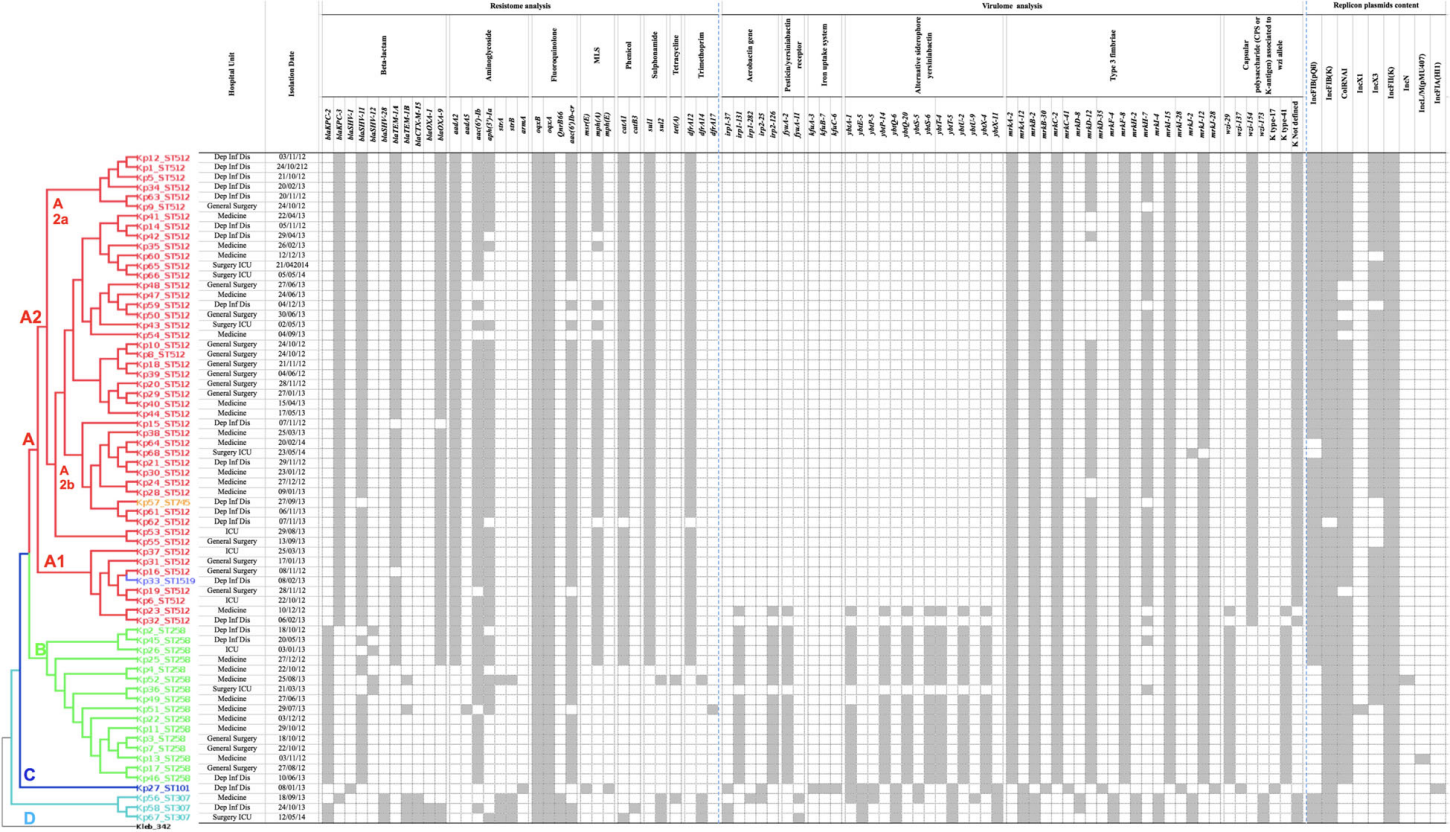
Core SNP tree, resistome, virulome and plasmid replicon analysis of 68 carbapenem-resistant *K. pneumoniae* strains. The maximum likelihood core SNP tree (left) was constructed by using *K. pneumoniae* 342 as reference genome. Major genetically distinct lineages are indicated by capital letters and branches labelled in different colours on the basis of the MLST type. The resistome, virulome and plasmid replicon analysis boxes (right) show the presence (grey) or absence (white) of the relevant genes for each of the isolates

The isolates’ final assembly obtained by means of WGS ranged from 96 to 237 contigs of >500 bps/sample, with N50 values of between 68,340 and 151,723, thus covering ~5.8 Mb of the *K. pneumoniae* genome. The molecular analysis was made in order to obtain information about the MLST, resistomes, virulomes, plasmid replicon types and core SNP genotypes of all of the strains (Fig. 2).

The MLST analysis, which was based on seven housekeeping genes (*rpoB, gapA, mdh, pgi, phoE, infB* and *tonB*), revealed six different sequence types, four of which (ST258, ST512, ST745 and ST1519) were members of clonal complex (CC) 258. ST258 was the “founding” CC genotype whose diversification was reflected in the appearance of the other STs: ST512 was a single-locus variant (SLV) of the ancestral ST, whereas ST745 and ST1519 were double-locus variants (DLVs). ST101 and ST307 were evolutionarily independent of the CC. The GoeBURST radial diagram (Additional file 2: Figure S1) shows the relatedness and patterns of evolutionary descent among the defined STs: the most representative were ST512 (46/68) and ST258 (16/68), whereas three strains belonged to ST307 and a further three were identified as ST101, ST745 and ST1519.

The in silico β-lactamase characterisation of the 68 sequenced isolates analysed 23 gene loci reported on the Pasteur website. The most frequent carbapenemase producing genes were *blaKPC* and *blaSHV* (both 98.53%), followed by *blaTEM* (80.88%), *blaCTX-M* (4.41%) and *blaOXA* (2.94%). The dissemination of *blaKPC* genes was sustained by the *blaKPC-2* variants (ST258 and two ST307 isolates) and the *blaKPC-3* allele (ST512 and their ST745 and ST1519 single-locus variants, and one ST307 isolate) (Fig. 2). Many *blaKPC* genes are associated with the Th4401 promiscuous transposon-related structure (which is approximately 10 kb in size and consists of a transposase, a resolvase, the *blaKPC* gene, and two insertion sequences, ISKpn6 and ISKpn7) contained in a number of different plasmids often belonging to incompatibility (Inc) group F [33]. BLAST alignments between the KPC-2 and KPC-3 contigs and the IncF Tn4401a pKpQIL-IT plasmid region (Plasmid Accession No. JN233705.2) showed that *blaKPC-2* and *blaKPC-3* genes were embedded in the Tn4401a transposon isoform.

The ST101 isolate (Kp27) was resistant to carbapenems in the absence of *blaKPC* genes and, in this isolate alone, the possible loss or alteration of outer membrane porins was investigated. GenBank accession numbers for *K. pneumoniae* strains used as reference for *OmpK35* and *OmpK36* gene sequences were AJ011501 and FJ577673, respectively. The *ompK35* sequence of Kp27 showed a perfect match of 1195/1202 bp with the reference, and a nucleotide deletion that leads to a truncated protein product of 63 amino acids. The *ompK36* sequence of Kp27 perfectly matched only 1098 of the 1151 *ompK36_*FJ577673 nucleotides and 20 bp gaps led to amino acid deletions and substitutions (see Kp27_ST101_omp35_omp36_sequences_analysis.doc in the Additional file 3).

In most of our isolates, the presence of β-lactamase genes was associated with sequences encoding for resistance to other classes of antibiotics: aminoglycosides (*aadA2, aadA5, aac(3′)-Ia, aac(6′)-Ib, strA, strB* and *armA* genes), fluoroquinolone (*oqxA, oqxB, qnrB66* and *aac(6′)-Ib-cr* genes), MLS (*msrE, mphA and mphE* genes), phenicol (*catA1* and *catB3* genes), sulfonamide (*sul1* and *sul2* genes), tetracycline (*tetA* gene) and trimethoprim (*dfrA-12/14/17* alleles) (Fig. 2).

In order to investigate AcrAB-TolC efflux pump-dependent and pump-independent tigecycline resistance mechanisms, we analysed the sequences of the *acrR*, *ramR*, *marR*, *soxR*, *lon*, *rpsJ* and *rpoC* genes [25, 26] by aligning our isolates with the reference genes of the tigecycline-susceptible isolate *K. pneumoniae* subsp. *pneumoniae* MGH78578 (GenBank Accession No. CP000647). The most frequently observed mutations were SNPs, but the gene sequences varied in terms of their presence, mutations and deletions. However, it was not possible to correlate these genomic features with tigecycline resistance because of the presence of a common mutation in susceptible, intermediate and resistant strains.

Many of the transcriptional regulation systems controlling lipopolysaccharide (LPS) modifications are involved in colistin resistance and, recently, the mobile *mcr-1* and *mcr-2* genes have been related to the increasing colistin resistance induced by the over-use of polymyxins in animals [27, 28]. We studied the *mgrB, pmrA/pmrB, phoP/phoQ* chromosomal genes taking the colistin-susceptible isolate *K. pneumoniae* subsp. *pneumoniae* MGH78578 (GenBank Accession No. CP000647) as reference, and also looked for the presence of plasmidic *mcr-1* (reference: *E. coli* pHNSHP45 Accession No. KP347127.1) and *mcr-2* genes (reference*: E. coli* KP37 pKP37-BE Accession No. NG_051171.1). The BLAST results revealed some changes in the chromosomal genes, and nucleotide variations were found in the *pmrA/pmrB* and *phoP/phoQ* genes of all of the colistin-resistant strains (Kp6, Kp19, Kp34, Kp37, Kp64, Kp65 and Kp68) and in colistin-sensitive isolates. Conversely, there wee some interesting variations in the *mgrB* gene in Kp19 and Kp37, with the locus being disrupted by an IS5-like element (best match: KKBO-4 isolate, Accession No. HG008893.1). Isolates Kp6, Kp64, Kp65 and Kp68 showed a wild-type *mgrB* gene, whereas the Kp34 *mgrB* contig started with the query sequence, and covered only 116/144 bps of the reference. The plasmid hits *mcr-1* and *mcr-2* were not found.

Figure 2 also shows the virulence repertoire detected in our samples. The *mrk* operon, which encodes type 3 fimbrial adhesins was detected in all of the isolates, and *mrkD* and *mrkH* genes were respectively missing from some ST512 and ST258 isolates. Aerobactin (*irp2*) genes, pesticin/yersiniabactin receptor (*fya*) genes, iron uptake system (*kfu*) genes, and alternative siderophore yersiniabactin (*ybt*) genes were detected in only one ST512 sample (Kp23), in all of the ST258 isolates except for Kp36, and in ST101 and ST307. Comparison of the genomes of our isolates with a reference *K. pneumoniae* strain (Kp13, GenBank Accession No. CP003999) showed that the virulence genes were located chromosomally. The iron acquisition systems were in a region of genomic plasticity consisting of a ~ 65 kbp integrative and conjugative element related to the *Yersinia pestis* high pathogenicity island (HPI), which is present in many *Enterobacteriaceae* genera (Additional file 4: Figure S2). The presence of genes coding for the yersiniabactin siderophores (*irp1* and *irp2*) are characteristic of this HPI, and their occurrence in more virulent *K. pneumoniae* strains has been previously reported [34]. Finally, the K-antigens related to the *wzi* alleles detected in our isolates were K type-41/*wzi-29* (all ST258 samples), K not defined/*wzi-154* or *wzi-173* (ST307 and ST512) and K type-17/*wzi-137* (ST101).

Using the PlasmidFinder web server, a total of nine plasmid replicon types were detected and characterised: IncFIB(pQil), IncFIB(K), ColRNAI, IncX1, IncX3, IncFII(K), IncN, IncL/M(pMU407 and IncFIA(HI1) (Accession Nos. of reference plasmids currently included in the Genbank database: JN233705, JN233704, DQ298019, EU370913, JN247852, CP000648, AY046276, U27345 and AF250878). The similarity in alignment between the best matching plasmid in the database and the corresponding sequence in the input genome ranged from 97.97% to 100%. Between three and five replicons were identified in each *K. pneumoniae* isolate and in various combinations: four arrangements of three plasmid replicons, five different combination of four replicon types, and two different combinations of five plasmid replicons (Fig. 2).

A maximum likelihood core SNP tree of the 68 *K. pneumoniae* isolates was constructed in order to provide high-resolution strain tracking and discrimination. Of the 38,120 SNPs identified, the 27,203 shared by all of the isolates could be divided into four major and genetically distinct lineages: A, B, C and D (Fig. 2). Lineage A had two major subgroups: A1 and A2. Subgroup A1 included seven ST512 isolates and the ST1519 isolate, whose resistome, virulome and plasmid content was almost identical, with the main exception of isolate Kp23 (ST512), which carried the *Yersinia pestis* HPI and belonged to the K type-41 group. The A2 isolates (*n* = 40) clustered into two major branches A2a (six ST512 isolates) and A2b (33 ST512 isolates and one ST745 isolate). A2a included identical strains, with the exception of the absence of the *mrkH-7* gene in isolate Kp9; the isolates in A2b were more variable and, interestingly, all of the strains lacking the fimbrial adhesin *mrkD* gene were in two separate sub-branches.

Lineage B had a series of six subgroups consisting of double or single branches (nine ST258 isolates), and included the strains with the most variable virulome, resistome and plasmid content. However, seven of these isolates, which were identical except for the presence of the plasmid replicons IncX1 in isolate Kp22 and IncL/M (pMU 407) in isolate Kp13, clustered together.

Lineage C included only the ST101 isolate, whereas lineage D (three ST307 isolates) could be divided into two subgroups on the basis of the resistome, virulome and plasmid replicon analyses, with the two closest isolates (containing the *blaOXA* genes and allele *blaKPC-2*) being grouped together.

The core SNP analysis was highly consistent with the MLST results and the resistome, virulome and plasmid profiles.

## Discussion

*Klebsiella pneumoniae* infection is an imminent threat to human health because of its virulence, its increasing resistance to antibiotics, and its ability to spread silently in human hosts who can act as carriers [34].

CRKP bacteria are some of the most challenging antibiotic-resistant pathogens involved in nosocomial infections worldwide because they greatly complicate the therapeutic management of hospitalised patients, thus leading to high morbidity and mortality rates, and also increasing hospital costs [35]. The global CRKP-related risk is even more worrying because of the growing number of strains that are resistant to last-resort drugs such as colistin and tigecycline [9, 10].

CRKP have rapidly become endemic in Italy, and there is a considerable likelihood of infectious outbreaks [36–38]. The relatedness of isolates during hospital outbreaks has traditionally been deduced on the basis of antimicrobial susceptibility patterns, crude microbiological methods, or molecular typing techniques such as REP-PCR and MLST, which has limited our knowledge of their genetic diversity within a single hospital [13].

WGS is becoming the gold standard in bacterial typing as it provides fine resolution and is highly discriminant in characterising isolates. Continuous technological advances, improvements in turnaround times, and the accessibility of DNA sequencing techniques are now approaching a stage at which genomic data can be rapidly generated in order to facilitate our understanding of the spread and evolution of infectious agents within a clinically useful time frame [39, 40].

The aim of this study was to analyse the alarming and prolonged outbreak of KRCP infection that occurred between January 2012 and December 2014 in different wards of Luigi Sacco University Hospital-ASST Fatebenefratelli Sacco, Milan, Italy. Traditional methods were used to investigate the antimicrobial susceptibility and the clonal relationships of the CRKP strains, and this was complemented by WGS data and the results of an in silico analysis that allowed MLST, the characterisation of resistome, virulome, plasmid content, and core SNP genotyping.

Antibiotic susceptibility testing of the CRKP strains circulating in our hospital showed the emergence of multi-drug resistance characterised by a worrying increase in tigecycline and colistin resistance. These data were confirmed by the in silico detection of the presence of genes conferring resistance to β-lactam antibiotics, aminoglycosides, fluoroquinolones, phenicol, sulfonamide, tetracyclines, trimethoprim and macrolide-lincosamide-streptogramin. In line with the findings of an Italian epidemiological survey, β-lactamase characterisation of our CRKP isolates showed the predominance of carbapenemase production, and that the most frequently detected variants of the *blaKPC* gene were *blaKPC-2* and *blaKPC-3*. The *blaSHV, blaTEM, blaCTX-M* and *blaOXA* genes were also detected. However, we were unable to establish any clear correlations between the genetic background and the most complicated multifactorial tigecycline and colistin resistance mechanisms. The observed SNPs and gene losses, mutations and deletions were not exclusive to the tigecycline-resistant strains as defined by microbiological methods, and determining whether the resistance was mutation related would have required complementary experiments and the evaluation of expression levels. Moreover, although comparative genomic analyses in Italy and Spain have demonstrated that colistin resistance in *K. pneumoniae* clinical isolates following exposure to colistin may be related to the insertional inactivation of the *mgrB* gene [17, 37], this was found in only two of our colistin-resistant isolates (Kp19 and Kp37).

The co-existence or co-evolution of antibiotic resistance and virulence factors is one of the most worrying possibilities as it could lead to the emergence of untreatable invasive *K. pneumoniae* infections. *K. pneumoniae* strains can produce virulence factors such as fimbrial adhesions, and capsule and iron uptake systems that enhance colonisation. The expression of adhesins is particularly important during the colonisation stage, when mechanical forces such as peristalsis and salivary secretions hamper bacterial invasion of the host [41]. Type 3 fimbriae have been identified as accessories mediating the formation of biofilms on biotic and abiotic surfaces (e.g. catheters), and antimicrobial drug resistance can increase the likelihood of bacterial cells existing in the biofilm by up to one thousand times [42]. The presence of type 3 fimbriae encoded by the *mrk* cluster in all of our clinical isolates (39.7% involving complicated urinary infections) supports the importance of this virulence trait in the pathogenesis and spread of CRKP.

Capsules can also play an important role in Klebsiella persistence inside and outside human hosts by resisting complement-mediated lysis or phagocytosis, and offering some protection against environmental desiccation; they may also have a neutralising effect on antibodies as a result of the release of excessive capsular material [41]. However, we did not detect the presence of any of the well-known hypervirulent capsular serotypes (i.e. K1, K2 and K5) in our isolates, which supports the view that multidrug-resistant strains and hypervirulent clonal complexes do not currently overlap [43].

Iron scavenging is important during infections because hosts have little free iron under physiological conditions [44]. Interestingly, our finding of the *Yersinia pestis* HPI in all but one of the ST258 isolates from the 12 patients with complicated urinary tract infections, and in one ST512 isolate from a patient with septicemia, suggests that these pathogens had adapted to their hosts very well. It should be noted that the chromosomal rather than plasmidic location of the virulence genes is a pathogenic strategy because chromosomal genes are retained by replication even in the absence of selective pressure, and this reduces the likelihood of the gene being lost to the bacterial population [18]. The pathogenicity of *K. pneumoniae* is further increased by the additional and easy acquisition of β-lactamase encoding genes although, ultimately, the successful evolution of the infection also greatly depends on a number of host-dependent factors [45].

Together with some specific characteristics of the bacterial strain, plasmid replicon content is currently used as a marker for bacterial typing during epidemiological investigations because it is the primary source of gene variability [46]. *K. pneumoniae* can have multiple replicon types, which have been classified into discrete incompatibility (inc) groups or families on the basis of the inability of closely related plasmids to stably propagate within the same bacterial strain [47]. Whole-genome sequencing using short-read technologies (e.g. Illumina) has become cheap and accessible, but some limitations relating to de novo assembly prevent the accurate reconstruction of the genomic context surrounding repeated sequences in plasmids. However, comparison of our 68 *Klebsiella* genomic sequences with those in the PlasmidFinder database allowed us to detect the plasmids often associated with antimicrobial resistance in clinically relevant bacterial pathogens and relate them to the sequences currently included in the database. The variability in plasmid structure and content among strains isolated in the same hospital suggests that additional plasmid types and arrangements may occur, and close surveillance is required.

The genotyping results of the in silico MLST analysis showed that, as in a number of other countries, the epidemic spread of CRKP in Italy is mainly due to hyper-epidemic clonal complex 258. However, increasing clone diversity has been reported in the literature [48], and our samples included three ST307 strains and one ST101, in addition to the ST512, ST258, ST745 and ST1519 strains.

The identification of the whole-genome core SNP set allowed us to obtain a higher-resolution strain tracking and discrimination than those provided by automated REP-PCR, which is currently used in our hospital to investigate bacteria clonal relationships. The REP-PCR and MLST results did not completely correlate: the REP-PCR group 2 and larger group 3 contained all of the ST258 strain and its variants ST745 and ST1519, but also ST512 (Kp7), ST307 (Kp56) and ST101 (Kp27). The phylogenetic core SNP tree showed that the four major lineages correlated with the MLST results. Among the isolates with the same ST, it was often possible to identify common genetic traits associated with virulence (the *Yersinia pestis* HPI in all but one ST258 strain) and antibiotic resistance (ST258_*blaKPC-2* and ST512_*blaKPC-3* isolates, and the common *mgrB* gene disruption in two ST512 colistin- resistant strains). However, the phylogenetic reconstruction obtained from the core genome SNPs, which correlated well with the genome background of related isolates, was not fully corroborated by conventional epidemiological data.

The effective tracing and tracking of outbreak isolates in order to control the spread of *K. pneumoniae* requires a detailed understanding of possible transmission routes. In healthcare settings, *Klebsiella* bacteria can spread as a result of person-to-person contact or, less frequently, environmental contamination. Unfortunately, the retrospective nature of this study means that it has some limitations in terms of the acquisition of the patients’ epidemiological and clinical data. For example, it was often impossible to find details concerning previous hospitalisations in other facilities or previous antibiotic treatments in the patients’ medical records, or establish the degree of environmental contamination during a patient’s stay in our hospital. Consequently, our analysis hypothesised possible patient-to-patient transmission events only on the basis of *bla*KPC gene correlations between the isolates of patients who were in the same ward for an overlapping period. In the A2a sub-lineage, the Kp1 and Kp5 samples were isolated from two patients in the infectious diseases ward within a period of just a few days. In the A2b sub-lineage, two ST512 isolates (Kp8 and Kp10) from patients simultaneously admitted to the general surgery ward, two isolates (Kp24 and Kp28) from patients in the medicine ward, and two isolates (Kp61 and Kp62) from patients in the infectious diseases ward were considered to be involved in person-to-person transmission. Two further strains (Kp3 and Kp7), which grouped closely in the B branch of the core SNP tree, were recovered from patients simultaneously staying in the surgery ward. These results only partially correlated with the REP-PCR data, which showed a very close genetic correlation only in the case of two isolate pairs (Kp8/Kp10 and Kp24/28).

## Conclusion

In conclusion, our findings show that whole-genome analysis can accurately reconstruct the molecular characterisation of isolates, including resistome, virulome and plasmid replicon content, and determine the phylogenetic relationships among strains circulating in a single hospital. The future clinical implementation of real-time sequencing could transform the surveillance and management of problematic nosocomial pathogens by overcoming the resolution limitations of analysing only a small portion of the genome. The latest techniques may also reduce working times and allow the prompt administration of the best treatment, thus avoiding the emergence of new antibiotic-resistant strains. Finally, it may be possible to identify the genetic variations underlying changes in bacterial virulence or tissue specificity, and genomic surveillance of these key phenotypic attributes may improve our understanding of the pathogenicity of multi-resistant agents and control its evolution.

## Additional files

Additional file 1: Table S1. MICs (μg/ml) of antimicrobial agents for carbapenem-resistant *K. pneumoniae* strains tested in this study. (XLS 31 kb)

Additional file 2: Figure S1. GoeBURST diagram showing a “population snapshot” of the 68 carbapenem-resistant *K. pneumoniae* strains. Each sequence type (ST) is represented by a circle whose size reflects the number of strains sharing the same ST, and the number 1 indicates a difference of only one locus between the closest STs. The numbers in the table show the corresponding housekeeping gene alleles for each ST, and colored are those for single and double locus variants of ST258 (ST512, ST745 and ST1519), the “founder” of CC258. (PDF 93 kb)

Additional file 3: Sequencing profiles of *ompK35* and *ompK36* of Kp27 strain ST101. (DOC 61 kb)

Additional file 4: Figure S2. Genome analysis of the *Yersinia pestis* high pathogenicity island in the 68 carbapenem-resistant *K. pneumoniae* strains . The Mauve multiple alignment between the Kp13 *K. pneumoniae* strain (GenBank accession No. CP003999) and our isolates evidenced the chromosomal location of a region of genomic plasticity related to that of the *Yersinia pestis* high pathogenecity island. (PNG 526 kb)

Additional file 5: Table S2. Sequence accession numbers of the carbapenem-resistant *K. pneumoniae* strains tested in this study (NCBI BioProject id: PRJNA 385863). (DOCX 13 kb)

## Abbreviations

CC: Clonal complex
CRE: Carbapenem-resistant Enterobacteriaceae
CRKP: Carbapenem-resistant *Klebsiella pneumoniae*
DLV: Double-locus variant
KPC: *Klebsiella pneumoniae* carbapenemase
MLST: Multilocus sequence typing
REP-PCR: Extra-genic palindromic polymerase chain reaction
SLV: Single-locus variant
SNP: Single nucleotide polymorphism
ST: Sequence type
WGS: Whole genome sequencing

## References

[1] Molton JS, Tambyah PA, Ang BS, Ling ML, Fisher DA (2013). The global spread of healthcare-associated multidrug-resistant bacteria: a perspective from Asia. Clin Infect Dis.

[2] Morrissey I, Hackel M, Badal R, Bouchillon S, Hawser S, Biedenbach D (2013). A review of ten years of the Study for Monitoring Antimicrobial Resistance Trends (SMART) from 2002 to 2011. Pharmaceuticals.

[3] van Duijn PJ, Dautzenberg MJ, Oostdijk EA (2011). Recent trends in antibiotic resistance in European ICUs. Curr Opin Crit Care.

[4] Tzouvelekis LS, Markogiannakis A, Psichogiou M, Tassios PT, Daikos GL (2012). Carbapenemases in Klebsiella pneumoniae and other Enterobacteriaceae: an evolving crisis of global dimensions. Clin Microbiol Rev.

[5] Bush K (2010). Alarming β-lactamase-mediated resistance in multidrug-resistant *Enterobacteriaceae*. Curr Opin Microbiol.

[6] Tijet N, Sheth PM, Lastovetska O, Chung C, Patel SN, Melano RG (2014). Molecular Characterization of *Klebsiella pneumoniae* Carbapenemase (KPC) - Producing Enterobacteriaceae in Ontario, Canada, 2008-2011. PLoS One.

[7] Mathers AJ, Stoesser N, Chai W, Carroll J, Barry K, Cherunvanky A (2017). Chromosomal integration of the *Klebsiella pneumoniae* carbapenemase gene, *bla*KPC, in *Klebsiella* species is elusive but not rare. Antimicrob Agents Chemother.

[8] Livermore DM, Warner M, Mushtaq S, Doumith M, Zhang J, Woodford N (2011). What remains against carbapenem-resistant *Enterobacteriaceae*? Evaluation of chloramphenicol, ciprofloxacin, colistin, fosfomycin, minocycline, nitrofurantoin, temocillin and tigecycline. Int J Antimicrob Agents.

[9] Weterings V, Zhou K, Rossen JW, van Stenis D, Thewessen E, Kluytmans J, Veenemans J (2015). An outbreak of colistin-resistant *Klebsiella pneumoniae* carbapenemase-producing *Klebsiella pneumoniae* in the Netherlands (July to December 2013), with inter-institutional spread. Eur J Clin Microbiol Infect Dis.

[10] Monaco M, Giani T, Raffone M, Arena F, Garcia-Fernandez A, Pollini S, Network EuSCAPE-Italy, Grundmann H, Pantosti A, Rossolini GM. Colistin resistance superimposed to endemic carbapenem- resistant *Klebsiella pneumoniae*: a rapidly evolving problem in Italy, November 2013 to April. Euro Surveill. 2014;19 (42). 10.2807/1560-7917.es2014.19.42.20939 25358041

[11] Palmore TN, Henderson DK (2013). Managing transmission of carbapenem-resistant enterobacteriaceae in healthcare settings: a view from the trenches. Clin Infect Dis.

[12] Giani T, Pini B, Arena F, Conte V, Bracco S, Migliavacca R, the AMCLI-CRE Survey Participants, Pantosti A, Pagani L, Luzzaro F, Rossolini GM. Epidemic diffusion of KPC carbapenemase-producing *Klebsiella pneumoniae* in Italy: results of the first countrywide survey, 15 May to 30 June 2011. Euro Surveill. 2013;18 (22). 23787077

[13] Sabat AJ, Budimir A, Nashev D (2013). ESCMID Study Group of Epidemiological Markers (ESGEM). Overview of molecular typing methods for outbreak detection and epidemiological surveillance. Euro Surveill.

[14] Köser CU, Ellington MJ, Peacock SJ (2014). Whole-genome sequencing to control antimicrobial resistance. Trends Genet.

[15] Lee Y, Kim BS, Chun J, Yong JH, Lee YS, Yoo JS (2014). Clonality and Resistome analysis of KPC-producing *Klebsiella pneumoniae* strain isolated in Korea using whole genome sequencing. Biomed Res Int.

[16] Zhao F, Bai J, Wu J, Liu J, Zhou M, Xia S (2010). Sequencing and genetic variation of multidrug resistance plasmids in *Klebsiella pneumoniae*. PLoS One.

[17] Ramirez MS, Traglia GM, Lin DL, Tran T, Tolmasky ME (2014). Plasmid-Mediated Antibiotic Resistance and Virulence in Gram-Negatives: the *Klebsiella pneumoniae* Paradigm. Microbiol Spectr.

[18] Mathers AJ, Peirano G, Pitout JD (2015). The role of epidemic resistance plasmids and international high-risk clones in the spread of multidrug-resistant *Enterobacteriaceae*. Clin Microbiol Rev.

[19] EUCAST (European Committee on Antimicrobial Susceptibility Testing), 2012; Breakpoint tables for interpretation of MICs and zone diameters. Version 2.0, valid from 2012–01-01. http://www.eucast.org/clinical_breakpoints/

[20] Andrews S. FastQC: a quality control tool for high throughput sequence data. 2010. https://www.bioinformatics.babraham.ac.uk/projects/fastqc/

[21] Joshi NA, Fass JN (2011). Sickle: a sliding-window, adaptive, quality-based trimming tool for FastQ files.

[22] Simpson JT, Kim Wong K, Shaun D, Jackman SD, Jacqueline E, Schein JE (2009). ABySS: a parallel assembler for short read sequence data. Genome Res.

[23] Francisco AP, Bugalho M, Ramirez M, Carrico JA (2009). Global Optimal eBURST analysis of Multilocus typing data using a graphic matroid approach. BMC Bioinformatics.

[24] Francisco AP, Vaz C, Monteiro PT, Melo-Cristino J, Ramirez M, Carriço JA (2012). PHYLOViZ: phylogenetic inference and data visualization for sequence based typing methods. BMC Bioinformatics.

[25] He F, Fu Y, Chen Q, Ruan Z, Hua X, Zhou H, Yu Y (2015). Tigecycline susceptibility and the role of efflux pumps in tigecycline resistance in KPC-producing Klebsiella pneumoniae. PLoS One.

[26] Fang L, Chen Q, Shi K, Li X, Shi Q, He F (2016). Step-wise increase in tigecycline resistance in Klebsiella pneumoniae associated with mutations in ramR, lon and rpsJ. PLoS One.

[27] Liu YY, Wang Y, Walsh TR, Yi LX, Zhang R, Spencer J (2016). Emergence of plasmid-mediated colistin resistance mechanism MCR-1 in animals and human beings in China: a microbiological and molecular biological study. Lancet Infect Dis.

[28] Xavier BB, Lammens C, Ruhal R, Kumar-Singh S, Butaye P, Goossens H, Malhotra-Kumar S. Identification of a novel plasmid-mediated colistin-resistance gene, mcr-2, in *Escherichia coli*, Belgium, June 2016. Euro Surveill. 2016;21(27). 10.2807/1560-7917.ES.2016.21.27.30280 27416987

[29] Choi Y, Chan A (2015). Provean web server: a tool to predict the functional effect of anninoacid sobstitution and indels. Bioinformatics.

[30] Darling AE, Mau B, Blattner FR, Perna NT (2004). Mauve: multiple alignment of conserved genomic sequence with rearrangements. Genome Res.

[31] Gardner SN, Hall BG (2013). When whole-genome alignments just won't work: kSNP v2 software for alignment-free SNP discovery and phylogenetics of hundreds of microbial genomes. PLoS One.

[32] Huson DH and Scornavacca C. Dendroscope 3: an interactive tool for rooted phylogenetic trees and networks, Syst. Biol*.* 2012; 61: 1061–7. (Software freely available at http://dendroscope.org). 10.1093/sysbio/sys062 22780991

[33] Lee C-R, Lee JH, Park KS, Kim YB, Jeong BC, Lee SH (2016). Global Dissemination of Carbapenemase-Producing Klebsiella pneumoniae: Epidemiology, Genetic Context, Treatment Options, and Detection Methods. Front Microbiol.

[34] Nordmann P, Cuzon G, Naas T (2009). The real threat of *Klebsiella pneumoniae* carbapenemase-producing bacteria. Lancet Infect Dis.

[35] Patel G, Huprikar S, Factor SH, Jenkins SG, Calfee DP (2008). Outcomes of carbapenem-resistant *Klebsiella pneumoniae* infection and the impact of antimicrobial and adjunctive therapies. Infect Control Hosp Epidemiol.

[36] Mammina C, Palma DM, Bonura C, Anna Plano MR, Monastero R, Sodano C (2010). Outbreak of infection with *Klebsiella pneumoniae* sequence type 258 producing *Klebsiella pneumoniae* Carbapenemase 3 in an intensive care unit in Italy. J Clin Microbiol.

[37] Giani T, Arena F, Vaggelli G, Conte V, Chiarelli A, Henrici De Angelis L (2015). Large nosocomial outbreak of colistin-resistant, carbapenemase-producing *Klebsiella pneumoniae* traced to clonal expansion of an mgrB deletion mutant. J Clin Microbiol.

[38] Ridolfo AL, Rimoldi SG, Pagani C, Marino AF, Piol A, Rimoldi M (2016). Diffusion and transmission of carbapenems-resistant *Klebsiella pneumoniae* in the medical and surgical wards of a university hospital in Milan, Italy. J Infect Public Health.

[39] Snitkin ES, Zelazny AM, Thomas PJ, Stock F; NISC Comparative Sequencing Program Group, Henderson DK, Palmore TN, Segre JA. Tracking a Hospital Outbreak of Carbapenem-Resistant *Klebsiella pneumoniae* with Whole-Genome Sequencing. Sci Transl Med. 2012;4:148ra116. 10.1126/scitranslmed.3004129 PMC3521604 22914622

[40] Onori R, Gaiarsa S, Comandatore F, Pongolini S, Brisse S, Colombo A (2015). Tracking nosocomial Klebsiella pneumoniae infections and outbreaks by whole genome analysis: small-scale Italian scenario within a single hospital. J Clin Microbiol.

[41] Wilson JW, Schurr MJ, LeBlanc CL, Ramamurthy R, Buchanan KL, Nickerson CA (2002). Mechanisms of bacterial pathogenicity. Postgrad Med J.

[42] Ong C-LY, Beatson SA, Totsika M, Forestier C, McEwan AG, Schembri MA (2010). Molecular analysis of type 3 fimbrial genes from Escherichia coli, Klebsiella and Citrobacter species. BMC Microbiol.

[43] Bialek-Davenet S, Criscuolo A, Ailloud F, Passet V, Jones L, Delannoy-Vieillard AS (2014). Genomic definition of hypervirulent and multidrug-resistant Klebsiella pneumoniae clonal groups. Emerg Infect Dis.

[44] Schaible UE, Kaufmann SHE (2004). Iron and microbial infection. Nat Rev.

[45] De Jesus MB, Ehlers MM, Dos Santos RF, Kock MM. Understanding β-lactamase producing Klebsiella pneumoniae. InTechOpen. 2015; doi:10.5772/61852.

[46] Carattoli A, Zankari E, Garcia-Fernandez A, Volby Larsen M, Lund O, Villa L (2014). PlasmidFinder and pMLST: in silico detection and typing of plasmids. Antimicrob Agents Chemother.

[47] Ramos PI, Picão RC, Almeida LG, Lima NC, Girardello R, Vivan AC (2014). Comparative analysis of the complete genome of KPC-2-producing *Klebsiella pneumoniae* Kp13 reveals remarkable genome plasticity and a wide repertoire of virulence and resistance mechanisms. BMC Genomics.

[48] Conte V, Monaco M, Giani T, D'Ancona F, Moro ML, Arena F (2016). AR-ISS Study Group on Carbapenemase-Producing K. pneumoniae. Molecular epidemiology of KPC-producing Klebsiella pneumoniae from invasive infections in Italy: increasing diversity with predominance of the ST512 clade II sublineage. J Antimicrob Chemother.

