# Supplementary material for: Whole genome sequencing for the molecular characterization of carbapenem-resistant Klebsiella pneumoniae strains isolated at the Italian ASST Fatebenefratelli Sacco Hospital, 2012–2014
Source: BMC Infect Dis. 2017 Oct 10;17:666. doi: 10.1186/s12879-017-2760-7 (PMC5634883; doi:10.1186/s12879-017-2760-7)
Supplement: Supplementary file 2 — GoeBURST diagram showing a “population snapshot” of the 68 carbapenem-resistant K. pneumoniae strains. Each sequence type (ST) is represented by a circle whose size reflects the number of strains sharing the same ST, and the number 1 indicates a difference of only one locus between the closest STs. The numbers in the table show the corresponding housekeeping gene alleles for each ST, and colored are those for single and double locus variants of ST258 (ST512, ST745 and ST1519), the “founder” of CC258. (PDF 93 kb) [file 12879_2017_2760_MOESM2_ESM.pdf]

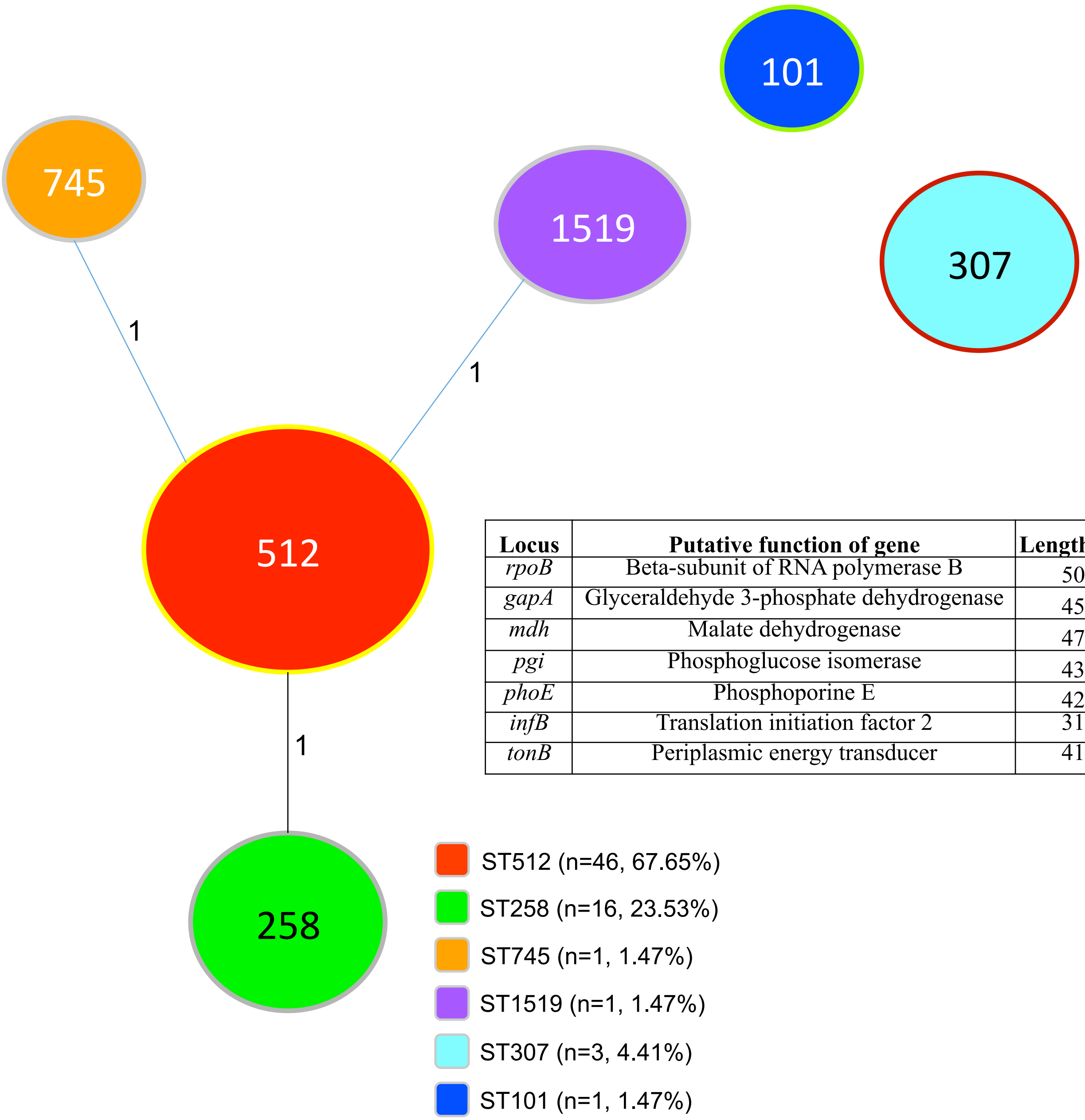

			**CC258**					
**Locus**	**Putative function of gene**	**Length (bp)**	**ST258**	**ST512**	**ST745**	**ST1519**	**ST101**	**ST307**
rpoB	Beta-subunit of RNA polymerase B	501	1	1	**67**	**9**	1	1
gapA	Glyceraldehyde 3-phosphate dehydrogenase	450	3	**54**	**54**	**54**	2	4
mdh	Malate dehydrogenase	477	1	1	1	1	1	2
pgi	Phosphoglucose isomerase	432	1	1	1	1	5	52
phoE	Phosphoporine E	420	1	1	1	1	4	1
infB	Translation initiation factor 2	318	3	3	3	3	6	1
tonB	Periplasmic energy transducer	414	79	79	79	79	6	7
