# Supplementary material for: Whole genome sequencing for the molecular characterization of carbapenem-resistant Klebsiella pneumoniae strains isolated at the Italian ASST Fatebenefratelli Sacco Hospital, 2012–2014
Source: BMC Infect Dis. 2017 Oct 10;17:666. doi: 10.1186/s12879-017-2760-7 (PMC5634883; doi:10.1186/s12879-017-2760-7)
Supplement: Supplementary file 3 — Sequencing profiles of ompK35 and ompK36 of Kp27 strain ST101. (DOC 61 kb) [file 12879_2017_2760_MOESM3_ESM.doc]

[**AJ011501.1**](https://www.ncbi.nlm.nih.gov/nucleotide/3650339?report=genbank&log$=nuclalign&blast_rank=90&RID=ASYRMAEA014) ***ompK35* protein**

MMKRNILAVVIPALLVAGAANAAEIYNKNGNKLDFYGKMVGEHVWTTNGDTSSDDTTYARIGLKGETQINDQLIGYGQWEYNMDASNVEGSQTTKTRLAFAGLKAGEYGSFDYGRNYGAIYDVEAATDMLVEWGGDGWNYTDNYMTGRTNGVATYRNSDFFGLVDGLSFALQYQGKNDHDRAIRKQNGDGFSTAATYAFDNGIALSAGYSSSNRSVDQKADGNGDKAEAWATSAKYDANNIYAAVMYSQTYNMTPEEDNHFAGKTQNFEAVVQYQFDFGLRPSIGYVQTKGKDLQSRAGFSGGDADLVKYIEVGTWYYFNKNMNVYAAYKFNQLDDNDYTKAAGVATDDQAAVGIVYQF

**Blast results of Query_Kp27_ST101 versus** [**AJ011501.1**](https://www.ncbi.nlm.nih.gov/nucleotide/3650339?report=genbank&log$=nuclalign&blast_rank=90&RID=ASYRMAEA014) ***ompK35* gene**

[**AJ011501.1**](https://www.ncbi.nlm.nih.gov/nucleotide/3650339?report=genbank&log$=nuclalign&blast_rank=90&RID=ASYRMAEA014) Klebsiella pneumoniae (strain KT755) ompK35 gene

Length=1202

Score = 2180 bits (1180), Expect = 0.0

Identities = 1195/1202 (99%), Gaps = 1/1202 (0%)

Strand=Plus/Plus

Query_**Kp27_ST101** 1 GGATGGAAAGATGCCTTCAGACACCAAACTCTCATCAATGGTTCTGTAAGTTTTTATTGA 60

||||||||||||||||||||||||||||||||||||||||||||||||||||||||||||

Sbjct 1 GGATGGAAAGATGCCTTCAGACACCAAACTCTCATCAATGGTTCTGTAAGTTTTTATTGA 60

Query 61 CAGAACTTATTGACGGCAGTGGCACGTGTTCATATAAAAAATATTAATGAGGGTAATAAA 120

||||||||||||||||||||||||||||||||||||||||||||||||||||||||||||

Sbjct 61 CAGAACTTATTGACGGCAGTGGCACGTGTTCATATAAAAAATATTAATGAGGGTAATAAA 120

CDS: Putative 1 1 M M K R N I L A V V I P A L L V A G A

Query 121 TAATGATGAAGCGCAATATTCTGGCAGTGGTGATCCCTGCCCTGCTGGTAGCCGGTGCAG 180

||||||||||||||||||||||||||||||||||||||||||||||||||||||||||||

Sbjct 121 TAATGATGAAGCGCAATATTCTGGCAGTGGTGATCCCTGCCCTGCTGGTAGCCGGTGCAG 180

CDS:OmpK35 porin [Kl 1 M M K R N I L A V V I P A L L V A G A

CDS: Putative 1 20 A N A A E I Y N K N G N K L D F Y G K M

Query 181 CCAACGCTGCAGAAATCTATAACAAAAACGGCAACAAACTGGACTTCTATGGAAAAATGG 240

||||||||||||||||||||||||||||||||||||||||||||||||||||||||||||

Sbjct 181 CCAACGCTGCAGAAATCTATAACAAAAACGGCAACAAACTGGACTTCTATGGAAAAATGG 240

CDS:OmpK35 porin [Kl 20 A N A A E I Y N K N G N K L D F Y G K M

CDS: Putative 1 40 V G E H V W T T N G D T S S D D T T Y A

Query 241 TCGGCGAGCACGTCTGGACCACCAATGGCGACACCAGTAGCGACGATACCACCTATGCCC 300

||||||||||||||||||||||||||||||||||||| ||||||||||||||||||||||

Sbjct 241 TCGGCGAGCACGTCTGGACCACCAATGGCGACACCAGCAGCGACGATACCACCTATGCCC 300

CDS:OmpK35 porin [Kl 40 V G E H V W T T N G D T S S D D T T Y A

CDS: Putative 1 60 R I A ***** K A K L R S T I S * S A T A S G

Query 301 GTATC-GCC**TGA**AAGGCGAAACTCAGATCAACGATCAGCTGATCGGCTACGGCCAGTGGG 359

||||| ||||||||||||||||||||||||||||||||||||||||||||||||||||||

Sbjct 301 GTATCGGCCTGAAAGGCGAAACTCAGATCAACGATCAGCTGATCGGCTACGGCCAGTGGG 360

CDS:OmpK35 porin [Kl 60 R I **G L K G E T Q I N D Q L I G Y G Q W**

CDS: Putative 1 78 N T T W T R P M L K V P R P Q K P V W R

Query 360 AATACAACATGGACGCGTCCAATGTTGAAGGTTCCCAGACCACAAAAACCCGTCTGGCGT 419

||||||||||||||||||||||||||||||||||||||||||||||||||||||||||||

Sbjct 361 AATACAACATGGACGCGTCCAATGTTGAAGGTTCCCAGACCACAAAAACCCGTCTGGCGT 420

CDS:OmpK35 porin [Kl 80 **E Y N M D A S N V E G S Q T T K T R L A**

CDS: Putative 1 98 S R A * K R A N T V H S T M A V T T A R

Query 420 TCGCGGGCCTGAAAGCGGGCGAATACGGTTCATTCGACTATGGCCGTAACTACGGCGCGA 479

|||| |||||||||||||||||||||||||||||||||||||||||||||||||||||||

Sbjct 421 TCGCAGGCCTGAAAGCGGGCGAATACGGTTCATTCGACTATGGCCGTAACTACGGCGCGA 480

CDS:OmpK35 porin [Kl 100 **F A G L K A G E Y G S F D Y G R N Y G A**

CDS: Putative 1 117 S T T S K R Q P I C W L N G A V T A G T

Query 480 TCTACGACGTCGAAGCGGCAACCGATATGCTGGTTGAATGGGGCGGTGACGGCTGGAACT 539

||||||||||||||||||||||||||||||||||||||||||||||||||||||||||||

Sbjct 481 TCTACGACGTCGAAGCGGCAACCGATATGCTGGTTGAATGGGGCGGTGACGGCTGGAACT 540

CDS:OmpK35 porin [Kl 120 **I Y D V E A A T D M L V E W G G D G W N**

CDS: Putative 1 137 I P T T T * P V V P T A S Q P T V T P T

Query 540 ATACCGACAACTACATGACCGGTCGTACCAACGGCGTCGCAACCTACCGTAACTCCGACT 599

| ||||||||||||||||||||||||||||||||||||||||||||||||||||| ||||

Sbjct 541 ACACCGACAACTACATGACCGGTCGTACCAACGGCGTCGCAACCTACCGTAACTCTGACT 600

CDS:OmpK35 porin [Kl 140 **Y T D N Y M T G R T N G V A T Y R N S D**

CDS: Putative 1 156 S S V W L T V * A S R C S T R V K T T M

Query 600 TCTTCGGTCTGGTTGACGGTCTGAGCTTCGCGCTGCAGTACCAGGGTAAAAACGACCATG 659

|||||||||||||||||||||||||||||||||||||||||||||||||||||||||| |

Sbjct 601 TCTTCGGTCTGGTTGACGGTCTGAGCTTCGCGCTGCAGTACCAGGGTAAAAACGACCACG 660

CDS:OmpK35 porin [Kl 160 **F F G L V D G L S F A L Q Y Q G K N D H**

CDS: Putative 1 175 T V R F A S R M A T A S A P Q P P T R S

Query 660 ACCGTGCGATTCGCAAGCAGAATGGCGACGGCTTCAGCACCGCAGCCACCTACGCGTTCG 719

||||||||||||||||||||||||||||||||||||||||||||||||||||||||||||

Sbjct 661 ACCGTGCGATTCGCAAGCAGAATGGCGACGGCTTCAGCACCGCAGCCACCTACGCGTTCG 720

CDS:OmpK35 porin [Kl 180 **D R A I R K Q N G D G F S T A A T Y A F**

CDS: Putative 1 195 T T V S H C L Q A T P A L T V A S I R K

Query 720 ACAACGGTATCGCACTGTCTGCAGGCTACTCCAGCTCTAACCGTAGCGTCGATCAGAAAG 779

||||||||||||||||||||||||||||||||||||||||||||||||||||||||||||

Sbjct 721 ACAACGGTATCGCACTGTCTGCAGGCTACTCCAGCTCTAACCGTAGCGTCGATCAGAAAG 780

CDS:OmpK35 porin [Kl 200 **D N G I A L S A G Y S S S N R S V D Q K**

CDS: Putative 1 215 L T A M A T K P K P G R P L Q N M T L T

Query 780 CTGACGGCAATGGCGACAAAGCCGAAGCCTGGGCGACCTCTGCAAAATATGACGCTAACA 839

||||||||||||||||||||||||||||||||||||||||||||||||||||||||||||

Sbjct 781 CTGACGGCAATGGCGACAAAGCCGAAGCCTGGGCGACCTCTGCAAAATATGACGCTAACA 840

CDS:OmpK35 porin [Kl 220 **A D G N G D K A E A W A T S A K Y D A N**

CDS: Putative 1 235 T S M R P S C T P R L T T * L R K K I T

Query 840 ACATCTATGCGGCCGTCATGTACTCCCAGACTTACAACATGACTCCGGAAGAAGATAACC 899

||||||||||||||||||||||||||||||||||||||||||||||||||||||||||||

Sbjct 841 ACATCTATGCGGCCGTCATGTACTCCCAGACTTACAACATGACTCCGGAAGAAGATAACC 900

CDS:OmpK35 porin [Kl 240 **N I Y A A V M Y S Q T Y N M T P E E D N**

CDS: Putative 1 254 T S P V K L R T L K Q L Y S I S L T S A

Query 900 ACTTCGCCGGTAAAACTCAGAACTTTGAAGCAGTTGTACAGTATCAGTTTGACTTCGGCC 959

||||||||||||||||||||||||||||||||||||||||||||||||||||||||||||

Sbjct 901 ACTTCGCCGGTAAAACTCAGAACTTTGAAGCAGTTGTACAGTATCAGTTTGACTTCGGCC 960

CDS:OmpK35 porin [Kl 260 **H F A G K T Q N F E A V V Q Y Q F D F G**

CDS: Putative 1 274 C V R P S A T Y R P K A R T C S R V L A

Query 960 TGCGTCCGTCCATCGGCTACGTACAGACCAAAGGCAAGGACCTGCAGTCGCGTGCTGGCT 1019

||||||||||||||||||||||||||||||||||||||||||||||||||||||||||||

Sbjct 961 TGCGTCCGTCCATCGGCTACGTACAGACCAAAGGCAAGGACCTGCAGTCGCGTGCTGGCT 1020

CDS:OmpK35 porin [Kl 280 **L R P S I G Y V Q T K G K D L Q S R A G**

CDS: Putative 1 294 S P A A M R I W L N T S K W V P G T T L

Query 1020 TCTCCGGCGGCGATGCGGATCTGGTTAAATACATCGAAGTGGGTACCTGGTACTACTTTA 1079

||||||||||||||||||||||||||||||||||||||||||||||||||||||||||||

Sbjct 1021 TCTCCGGCGGCGATGCGGATCTGGTTAAATACATCGAAGTGGGTACCTGGTACTACTTTA 1080

CDS:OmpK35 porin [Kl 300 **F S G G D A D L V K Y I E V G T W Y Y F**

CDS: Putative 1 314 T R T * T S T L R I N S T S W T T T I T

Query 1080 ACAAGAACATGAACGTCTACGCTGCGTATAAATTCAACCAGCTGGACGACAACGATTACA 1139

||||||||||||||||||||||||||||||||||||||||||||||||||||||||||||

Sbjct 1081 ACAAGAACATGAACGTCTACGCTGCGTATAAATTCAACCAGCTGGACGACAACGATTACA 1140

CDS:OmpK35 porin [Kl 320 **N K N M N V Y A A Y K F N Q L D D N D Y**

CDS: Putative 1 333 P K R L V S P L T T R R P W V S F T S S

Query 1140 CCAAAGCGGCTGGTGTCGCCACTGACGACCAGGCGGCCGTGGGTATCGTTTACCAGTTCT 1199

||||||||||||||||||||||||||||||||||||||||||||||||||||||| ||||

Sbjct 1141 CCAAAGCGGCTGGTGTCGCCACTGACGACCAGGCGGCCGTGGGTATCGTTTACCAATTCT 1200

CDS:OmpK35 porin [Kl 340 **T K A A G V A T D D Q A A V G I V Y Q F**

Query 1200 AA 1201

||

Sbjct 1201 AA 1202

[**FJ577673.1**](https://www.ncbi.nlm.nih.gov/nucleotide/221192795?report=genbank&log$=nuclalign&blast_rank=74&RID=CACAETN5016) ***ompK36* protein**

MKVKVLSLLVPALLVAGAANAAEIYNKDGNKLDLYGKIDGLHYFSDDKSVDGDQTYMRVGVKGETQINDQLTGYGQWEYNVQANNTESSSDQAWTRLAFAGLKFGDAGSFDYGRNYGVVYDVTSWTDVLPEFGGDTYGSDNFLQSRANGVATYRNSDFFGLVDGLNFALQYQGKNGSVSGEGA**LSP**TNNGRTALKQNGDGYGTSLTYDIYDGISAGFAYSNSKRLGDQNSKLALGRGDNAETYTGGLKYDANNIYLATQYTQTYNATRAGSLGFANKAQNFEVVAQYQFDFGLRPSVAYLQSKGKDLEGYGDQDILKYVDVGATYYFNKNMSTYVDYKINLLDDNSFTHNAGISTDDVVALGLVYQ

**Blast results of Query_Kp27_ST101 versus** [**FJ577673.1**](https://www.ncbi.nlm.nih.gov/nucleotide/221192795?report=genbank&log$=nuclalign&blast_rank=74&RID=CACAETN5016) ***ompK36* gene**

[**FJ577673.1**](https://www.ncbi.nlm.nih.gov/nucleotide/3650339?report=genbank&log$=nuclalign&blast_rank=90&RID=ASYRMAEA014) Klebsiella pneumoniae (strain VM522) ompK36 gene

partial cds

Length=1144

Score = 1814 bits (982), Expect = 0.0

Identities = 1098/1151 (95%), Gaps = 20/1151 (2%)

Strand=Plus/Plus

CDS: Putative 1 1 M K V K V

Query 1 GCAGTGGCATAATAAAAGGCATATAACAAACAGAGGGTTAATAACATGAAAGTTAAAGTA 60

||||||||||||||||||||||||||||||||||||||||||||||||||||||||||||

Sbjct 1 GCAGTGGCATAATAAAAGGCATATAACAAACAGAGGGTTAATAACATGAAAGTTAAAGTA 60

CDS:OmpK36 porin, pa 1 M K V K V

CDS: Putative 1 6 L S L L V P A L L V A G A A N A A E I Y

Query 61 CTGTCCCTCCTGGTACCGGCTCTGCTGGTAGCAGGCGCAGCAAATGCGGCTGAAATTTAT 120

||||||||||||||||||||||||||||||||||||||||||||||||||||||||||||

Sbjct 61 CTGTCCCTCCTGGTACCGGCTCTGCTGGTAGCAGGCGCAGCAAATGCGGCTGAAATTTAT 120

CDS:OmpK36 porin, pa 6 L S L L V P A L L V A G A A N A A E I Y

CDS: Putative 1 26 N K D G N K L D L Y G K I D G L H Y F S

Query 121 AACAAAGACGGCAACAAATTAGACCTGTACGGTAAAATTGACGGTCTGCACTACTTCTCT 180

||||||||||||||||||||||||||||||||||||||||||||||||||||||||||||

Sbjct 121 AACAAAGACGGCAACAAATTAGACCTGTACGGTAAAATTGACGGTCTGCACTACTTCTCT 180

CDS:OmpK36 porin, pa 26 N K D G N K L D L Y G K I D G L H Y F S

CDS: Putative 1 46 D D K S V D G D Q T Y M R V G V K G E T

Query 181 GACGACAAGAGCGTCGACGGCGACCAGACCTACATGCGTGTAGGCGTGAAAGGCGAAACC 240

||||||||||||||||||||||||||||||||||||||||||||||||||||||||||||

Sbjct 181 GACGACAAGAGCGTCGACGGCGACCAGACCTACATGCGTGTAGGCGTGAAAGGCGAAACC 240

CDS:OmpK36 porin, pa 46 D D K S V D G D Q T Y M R V G V K G E T

CDS: Putative 1 66 Q I N D Q L T G Y G Q W E Y N V Q A N N

Query 241 CAGATCAACGACCAGCTGACCGGTTACGGCCAGTGGGAATACAACGTTCAGGCGAACAAC 300

||||||||||||||||||||||||||||||||||||||||||||||||||||||||||||

Sbjct 241 CAGATCAACGACCAGCTGACCGGTTACGGCCAGTGGGAATACAACGTTCAGGCGAACAAC 300

CDS:OmpK36 porin, pa 66 Q I N D Q L T G Y G Q W E Y N V Q A N N

CDS: Putative 1 86 T E S S S D Q A W T R L A F A G L K F G

Query 301 ACTGAAAGCTCCAGCGATCAGGCATGGACTCGTCTGGCATTCGCAGGCCTGAAATTTGGC 360

||||||||||||||||||||||||||||||||||||||||||||||||||||||||||||

Sbjct 301 ACTGAAAGCTCCAGCGATCAGGCATGGACTCGTCTGGCATTCGCAGGCCTGAAATTTGGC 360

CDS:OmpK36 porin, pa 86 T E S S S D Q A W T R L A F A G L K F G

CDS: Putative 1 106 D A G S F D Y G R N Y G V V Y D V T S W

Query 361 GACGCGGGCTCTTTCGACTACGGTCGTAACTACGGCGTAGTATACGACGTAACGTCCTGG 420

||||||||||||||||||||||||||||||||||||||||||||||||||||||||||||

Sbjct 361 GACGCGGGCTCTTTCGACTACGGTCGTAACTACGGCGTAGTATACGACGTAACGTCCTGG 420

CDS:OmpK36 porin, pa 106 D A G S F D Y G R N Y G V V Y D V T S W

CDS: Putative 1 126 T D V L P E F G G D T Y G S D N F L Q S

Query 421 ACCGACGTTCTGCCGGAATTCGGCGGCGACACCTACGGTTCTGACAACTTCCTGCAGTCC 480

||||||||||||||||||||||||||||||||||||||||||||||||||||||||||||

Sbjct 421 ACCGACGTTCTGCCGGAATTCGGCGGCGACACCTACGGTTCTGACAACTTCCTGCAGTCC 480

CDS:OmpK36 porin, pa 126 T D V L P E F G G D T Y G S D N F L Q S

CDS: Putative 1 146 R A N G V A T Y R N S D F F G L V D G L

Query 481 CGTGCTAACGGCGTTGCAACCTACCGTAACTCTGATTTCTTCGGTCTGGTTGACGGCCTG 540

||||||||||||||||||||||||||||||||||||||||||||||||||||||||||||

Sbjct 481 CGTGCTAACGGCGTTGCAACCTACCGTAACTCTGATTTCTTCGGTCTGGTTGACGGCCTG 540

CDS:OmpK36 porin, pa 146 R A N G V A T Y R N S D F F G L V D G L

CDS: Putative 1 166 N F A L Q Y Q G K N G S V S G E G A

Query 541 AACTTTGCTCTGCAGTATCAGGGTAAAAACGGCAGCGTCAGCGGCGAAGGCGC---G--- 594

||||||||||||||||||||||||||||||||||||||||||||||||||||| |

Sbjct 541 AACTTTGCTCTGCAGTATCAGGGTAAAAACGGCAGCGTCAGCGGCGAAGGCGCTCTGTCT 600

CDS:OmpK36 porin, pa 166 N F A L Q Y Q G K N G S V S G E G A L S

CDS: Putative 1 184 T N N G R G W S K Q N G D G F G T S

Query 595 ---ACCAACAACGGTCGTG--G--TTGGAGCAAACAGAACGGCGACGGCTTCGGCACCTC 647

||||||||||||||| | ||| ||||||||||||||||| | ||| || ||

Sbjct 601 CCTACCAACAACGGTCGTACCGCCTTG----AAACAGAACGGCGACGGTTACGGTACTTC 656

CDS:OmpK36 porin, pa 186 **P** T N N G R **T A L** K Q N G D G **Y** G T S

CDS: Putative 1 202 L T Y D I W D G I S A G F A Y S H S K R

Query 648 TCTGACCTACGATATTTGGGATGGCATCAGCGCTGGTTTCGCGTACTCTCACTCCAAACG 707

||||||||| || || | ||||||||||||||||||||||| |||||| ||||||||||

Sbjct 657 TCTGACCTATGACATCTATGATGGCATCAGCGCTGGTTTCGCATACTCTAACTCCAAACG 716

CDS:OmpK36 porin, pa 205 L T Y D I **Y** D G I S A G F A Y S **N** S K R

CDS: Putative 1 222 T D E Q N S V P A L G R G D N A E T Y T

Query 708 TACCGACGAGCAGAATAGTGTTCCGGCACTGGGTCGTGGCGACAACGCTGAAACCTACAC 767

| | ||| ||||| || | ||||||||||||||||||||||||||||||||||||

Sbjct 717 TCTTGGCGACCAGAACAGCAAGCTGGCACTGGGTCGTGGCGACAACGCTGAAACCTACAC 776

CDS:OmpK36 porin, pa 225 **L G D** Q N S **K L** A L G R G D N A E T Y T

CDS: Putative 1 242 G G L K Y D A N N I Y L A S Q Y T Q T Y

Query 768 CGGTGGTCTGAAATACGACGCCAACAACATCTACCTGGCCTCTCAGTACACCCAGACCTA 827

||| ||||||||||||||||| |||||||||||||||||| |||||||||||||||||||

Sbjct 777 CGGCGGTCTGAAATACGACGCGAACAACATCTACCTGGCCACTCAGTACACCCAGACCTA 836

CDS:OmpK36 porin, pa 245 G G L K Y D A N N I Y L A **T** Q Y T Q T Y

CDS: Putative 1 262 N A T R A G S L G F A N K A Q N F E V V

Query 828 CAACGCAACTCGCGCCGGTTCCCTGGGCTTTGCAAACAAAGCGCAGAACTTCGAAGTGGT 887

|||||| || ||||||||||||||||||||||| ||||||||||||||||||||||||||

Sbjct 837 CAACGCGACCCGCGCCGGTTCCCTGGGCTTTGCTAACAAAGCGCAGAACTTCGAAGTGGT 896

CDS:OmpK36 porin, pa 265 N A T R A G S L G F A N K A Q N F E V V

CDS: Putative 1 282 A Q Y Q F D F G L R P S V A Y L Q S K G

Query 888 TGCTCAGTACCAGTTCGACTTCGGTCTGCGTCCGTCTGTGGCTTACCTGCAGTCTAAAGG 947

|||||||||||||||||||||||||||||||||||| |||||||||||||||||||||||

Sbjct 897 TGCTCAGTACCAGTTCGACTTCGGTCTGCGTCCGTCCGTGGCTTACCTGCAGTCTAAAGG 956

CDS:OmpK36 porin, pa 285 A Q Y Q F D F G L R P S V A Y L Q S K G

CDS: Putative 1 302 K D L E R G Y G D Q D I L K Y V D V G A

Query 948 TAAGGATCTGGAGCGCGGCTACGGCGACCAGGACATCCTGAAATATGTTGACGTTGGCGC 1007

|||||||||||| ||||||||||||||||||||||||||||||||||||||||||||

Sbjct 957 TAAGGATCTGGAA---GGCTACGGCGACCAGGACATCCTGAAATATGTTGACGTTGGCGC 1013

CDS:OmpK36 porin, pa 305 K D L E G Y G D Q D I L K Y V D V G A

CDS: Putative 1 322 T Y Y F N K N M S T Y V D Y K I N L L D

Query 1008 GACCTACTACTTCAACAAAAACATGTCCACCTATGTTGACTACAAAATCAACCTGCTGGA 1067

||||||||||||||||||||||||||||||||||||||||||||||||||||||||||||

Sbjct 1014 GACCTACTACTTCAACAAAAACATGTCCACCTATGTTGACTACAAAATCAACCTGCTGGA 1073

CDS:OmpK36 porin, pa 324 T Y Y F N K N M S T Y V D Y K I N L L D

CDS: Putative 1 342 D N S F T R N A G I S T D D V V A L G L

Query 1068 CGACAACAGCTTCACCCGCAACGCCGGTATCTCTACCGACGACGTGGTTGCACTGGGCCT 1127

|||||| |||||||||| ||||||||||||||||||||||||||||||||||||||||||

Sbjct 1074 CGACAATAGCTTCACCCACAACGCCGGTATCTCTACCGACGACGTGGTTGCACTGGGCCT 1133

CDS:OmpK36 porin, pa 344 D N S F T **H** N A G I S T D D V V A L G L

CDS: Putative 1 362 V Y Q

Query 1128 GGTTTACCAGT 1138

|||||||||||

Sbjct 1134 GGTTTACCAGT 1144

CDS:OmpK36 porin, pa 364 V Y Q
