# Supplementary figures and images for: Whole genome sequencing for the molecular characterization of carbapenem-resistant Klebsiella pneumoniae strains isolated at the Italian ASST Fatebenefratelli Sacco Hospital, 2012–2014
Source: BMC Infect Dis. 2017 Oct 10;17:666. doi: 10.1186/s12879-017-2760-7 (PMC5634883; doi:10.1186/s12879-017-2760-7)

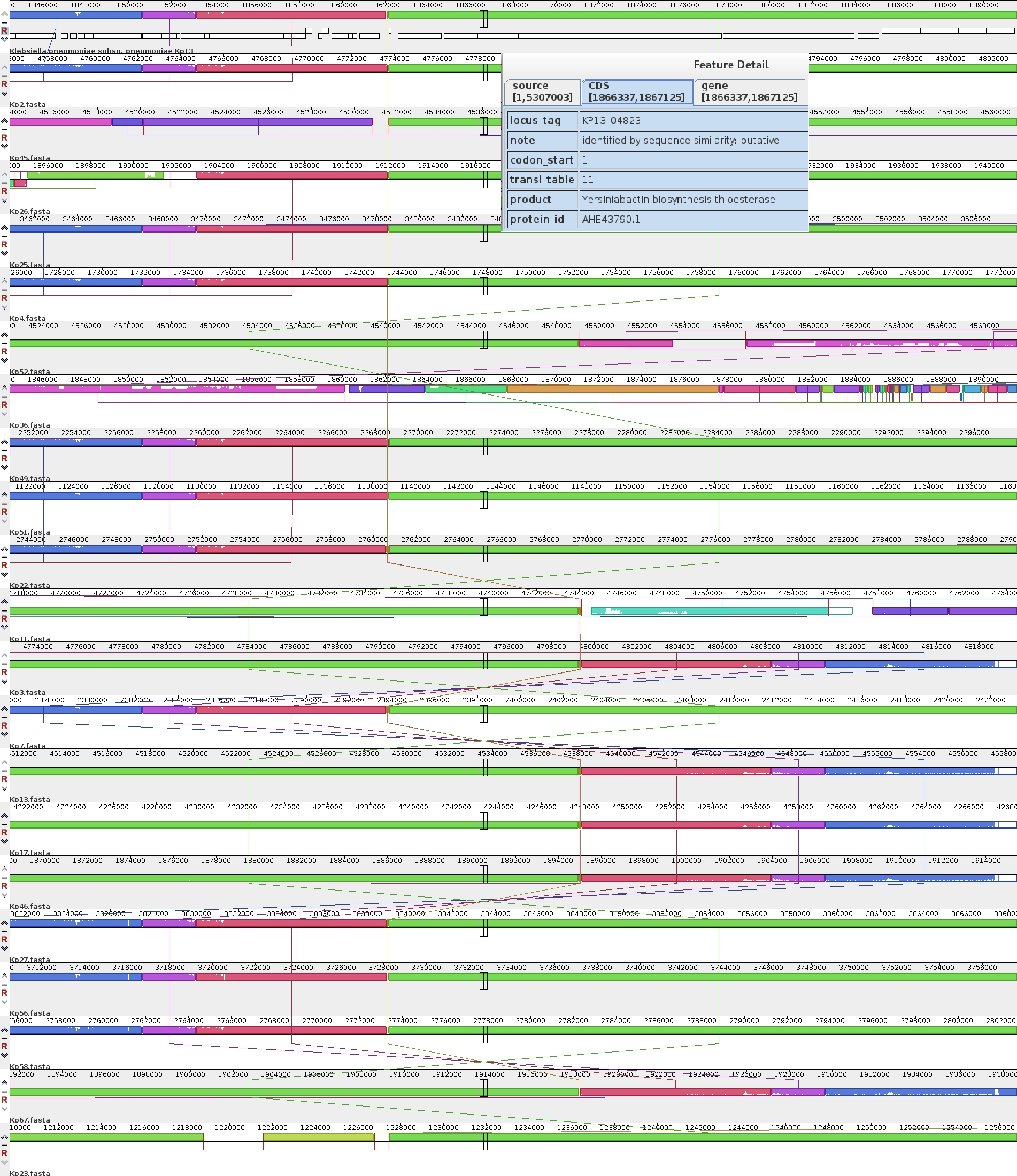

Supplement: Supplementary file 4 — Genome analysis of the Yersinia pestis high pathogenicity island in the 68 carbapenem-resistant K. pneumoniae strains . The Mauve multiple alignment between the Kp13 K. pneumoniae strain (GenBank accession No. CP003999) and our isolates evidenced the chromosomal location of a region of genomic plasticity related to that of the Yersinia pestis high pathogenecity island. (PNG 526 kb) [file 12879_2017_2760_MOESM4_ESM.png]
