# Supplementary material for: Whole genome sequencing for the molecular characterization of carbapenem-resistant Klebsiella pneumoniae strains isolated at the Italian ASST Fatebenefratelli Sacco Hospital, 2012–2014
Source: BMC Infect Dis. 2017 Oct 10;17:666. doi: 10.1186/s12879-017-2760-7 (PMC5634883; doi:10.1186/s12879-017-2760-7)
Supplement: Supplementary file 5 — Sequence accession numbers of the carbapenem-resistant K. pneumoniae strains tested in this study (NCBI BioProject id: PRJNA 385863). (DOCX 13 kb) [file 12879_2017_2760_MOESM5_ESM.docx]

| **Strain** | **Sequencing accession** |
| --- | --- |
| KP1 | SAMN06917594 |
| KP2 | SAMN06917595 |
| KP3 | SAMN06917596 |
| KP4 | SAMN06917597 |
| KP5 | SAMN06917598 |
| KP6 | SAMN06917599 |
| KP7 | SAMN06917600 |
| KP8 | SAMN06917601 |
| KP9 | SAMN06917602 |
| KP10 | SAMN06917603 |
| KP11 | SAMN06917604 |
| KP12 | SAMN06917605 |
| KP13 | SAMN06917606 |
| KP14 | SAMN06917607 |
| KP15 | SAMN06917608 |
| KP16 | SAMN06917609 |
| KP17 | SAMN06917610 |
| KP18 | SAMN06917611 |
| KP19 | SAMN06917612 |
| KP20 | SAMN06917613 |
| KP21 | SAMN06917614 |
| KP22 | SAMN06917615 |
| KP23 | SAMN06917616 |
| KP24 | SAMN06917617 |
| KP25 | SAMN06917618 |
| KP26 | SAMN06917619 |
| KP27 | SAMN06917620 |
| KP28 | SAMN06917621 |
| KP29 | SAMN06917622 |
| KP30 | SAMN06917623 |
| KP31 | SAMN06917624 |
| KP32 | SAMN06917625 |
| KP33 | SAMN06917626 |
| KP34 | SAMN06917627 |
| KP35 | SAMN06917628 |
| KP36 | SAMN06917629 |
| KP37 | SAMN06917630 |
| KP38 | SAMN06917631 |
| KP39 | SAMN06917632 |
| KP40 | SAMN06917633 |
| KP41 | SAMN06917634 |
| KP42 | SAMN06917635 |
| KP43 | SAMN06917636 |
| KP44 | SAMN06917637 |
| KP45 | SAMN06917638 |
| KP46 | SAMN06917639 |
| KP47 | SAMN06917640 |
| KP48 | SAMN06917641 |
| KP49 | SAMN06917642 |
| KP50 | SAMN06917643 |
| KP51 | SAMN06917644 |
| KP52 | SAMN06917645 |
| KP53 | SAMN06917646 |
| KP54 | SAMN06917647 |
| KP55 | SAMN06917648 |
| KP56 | SAMN06917649 |
| KP57 | SAMN06917650 |
| KP58 | SAMN06917651 |
| KP59 | SAMN06917652 |
| KP60 | SAMN06917653 |
| KP61 | SAMN06917654 |
| KP62 | SAMN06917655 |
| KP63 | SAMN06917656 |
| KP64 | SAMN06917657 |
| KP65 | SAMN06917658 |
| KP66 | SAMN06917659 |
| KP67 | SAMN06917660 |
| KP68 | SAMN06917661 |

                                                                                                    

                         

                         
